# Study of the mining and aquifer interactions in complex geological conditions and its management

**DOI:** 10.1038/s41598-023-34947-6

**Published:** 2023-06-10

**Authors:** Wanpeng Huang, Le Sui, Yanmin Wang, Chengguo Zhang, Donghai Jiang, Xianwei Cai, Zhixiang Yang

**Affiliations:** 1 College of Energy and Mining Engineering, Shandong University of Science and Technology, Qingdao, 266590 China; 2 State Key Laboratory of Efficient Mining and Clean Utilization of Coal Resource, Beijing, 100013 China; 3 Xiaoyun Coal Mine, Jining Energy Development Group Co. Ltd., Jining, 272000 China; 4 School of Minerals and Energy Resources Engineering, University of New South Wales, Sydney, NSW 2052 Australia

**Keywords:** Hydrology, Energy infrastructure

## Abstract

The interaction of mining and the surface water or aquifer system in varying overburden strata conditions is one of the most critical aspects of sustainable mining practices, that can lead to water loss or water inrush into openings. This paper examined this phenomenon in a complex strata condition via a case study, and proposed a new mining design to minimize the impact of longwall mining on the overlaying aquifer. A range of factors have been identified contributing to the potential disturbance of the aquifer, including the extent of the water-rich area, the characteristics of overburden rock units, and the development height of the water-conducting fracture zone. In this study, the transient electromagnetic method and the high-density three-dimensional electrical method were used to identify two areas prone to water inrush danger in the working face. The vertical range of the water-rich abnormal area 1 is 45–60 m away from the roof, with an area of 3334 m^2^. The vertical range of the water-rich abnormal area 2 is 30–60 m away from the roof, with an area of approximately 2913 m^2^. The bedrock drilling method was used to determine that the thinnest part of the bedrock, with a thickness of approximately 60 m, and the thickest part, with a thickness of approximately 180 m. The maximum mining-induced height of the fracture zone was 42.64 m using empirical method, theoretical prediction based on the rock stratum group, field monitoring. In summary, the high risk area was determined, and the analysis shows that the size of the water prevention) pillar was 52.6 m, which was smaller than the safe water prevention pillar actually set in the mining range. The research conclusion provides important safety guidance significance for the mining of similar mines.

## Introduction

Coal mining under the water body includes coal mining under the surface water body, coal mining under the loose aquifer water body and coal mining under the bedrock aquifer water body^1,2^. When the water body is a bedrock aquifer, the thickness of the bedrock is uneven, and the roof of the working face is covered with an aquifer. At positions with thin bedrock thicknesses, if the fracture zone formed after mining of the working face leads to an aquifer, there is a risk of water inrush disasters^3–6^.

The safe mining under the aquifer of the bedrock working face is affected by many factors, such as the scope of the water-rich area, the thickness and structure of the cover rock, and the development height of the water-conducting fracture zone^7–13^. Many researchers have relatively mature research in theory and practical application. Wang et al. predicted the height of the water-conducting fracture zone under different mining thickness conditions by establishing mechanical models^14–16^. Li et al. predicted and analyzed the feasibility of safe mining under aquifers based on mechanical theory and determined the critical conditions and prediction formulas for the occurrence of water inrush and sand bursting^17,18^. Chen et al. simulated the experimental process of water inrush and sand bursting disasters through similar materials and put forward feasible suggestions for safe mining under aquifers^19,20^. Yang Bin et al. described the complex nonlinear relationship between the index system under the aquifer and mining safety through mathematical models^21^. In summary, numerous researchers have performed many analyses on the current research situation of theories and technologies related to water control and obtained some important research results^22–24^. The above research has played an important role in guiding the feasibility analysis of safe mining under the theoretically predicted working face.

To ensure the safe mining of the working face under the complex bedrock aquifer, determining the danger range of the aquifer and the bedrock structure and predicting the development height of the fracture zone after the mining of the working face are the key problems to be solved first. Currently, the main way to determine the geological and hydrological work of mine exploration is still the field detection method, which is also the most direct and reliable method, including the drilling method, geochemical prospecting method, geophysical exploration methods (including high-density resistivity method, microseismic method, acoustic wave method), hydrogeological experiments, etc. The use of geophysical exploration methods to detect the scope of the water-rich area of the roof and the use of drilling methods to detect the thickness and structure of the cover rock can be more intuitive and accurate to determine the feasibility of safe mining under aquifers of complex bedrock working faces.

The 1318 comprehensive mining working face of Xiaoyun Coal Mine of Shandong Jining Mining Group has an irregular trapezoidal and pseudo-oblique layout. The thickness of the loose layer in the overlying strata layer is large, reflecting the unstable occurrence of bedrock, which is thick in some places and thin in some places, and the roof of the working face is covered with an aquifer. If the fractured zone formed after mining of the working face leads to the aquifer, there is a risk of a water inrush disaster. In this study, geophysical exploration methods were proposed to explore the scope of the water-rich area. The cover rock thickness and structure were detected by the drilling method. The height of the water-conducting fracture zone was predicted by empirical formula prediction, theoretical prediction, field detection analysis and other methods. Finally, it was judged whether the size of the water prevention coal (rock) pillar of the working face was in line with the safe mining range, and the feasibility of safe mining under the aquifer of the bedrock working face was comprehensively analyzed. The research conclusion provides important safety guidance significance for the mining of similar mines^25,26^.

## Project overview

**Figure 1:**
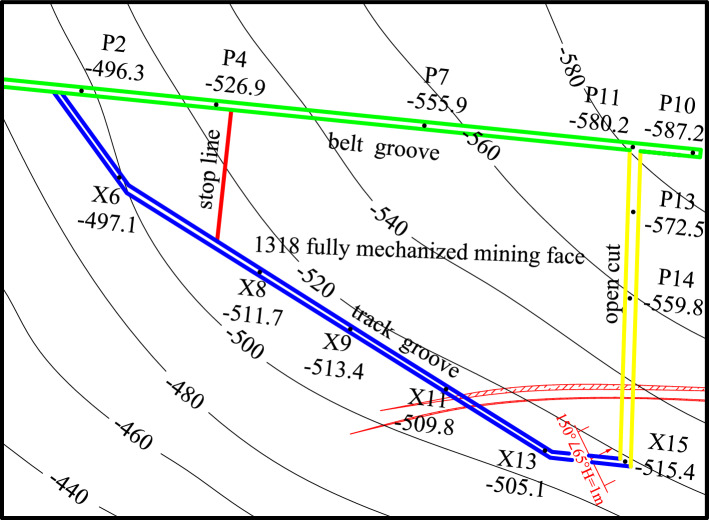
The 1318 Working face layout.

**Figure 2:**
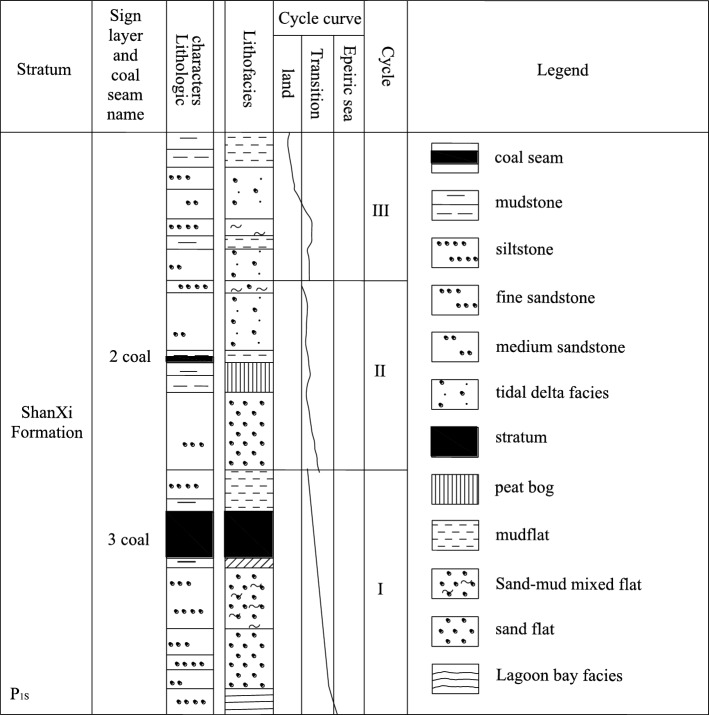
The 1318 Comprehensive geological histogram.

The Xiaoyun Coal Mine is located in Jining City, Shandong Province,China. Coal seam 3 is mainly mined. The coal seam strikes northwest and tends northeast. The dip angle of the coal seam is 13° ~ 26°, with an average of 17°. The average thickness of the coal seam is 2.8 m. As shown in Fig. 1. The strike of the working face is nearly east to west, and the tendency is nearly north in an irregular trapezoidal shape with a pseudo-oblique layout. The length of the working face at the cuthole is 220 m. With the advance of the working face, the length of the working face gradually shortens, the length of the working face at the stopping line is approximately 100 m, and the advancing length of the working face is 290 m. The roof mainly consists of various medium sandstone and fine sandstone, among which the immediate roof is fine sandstone with an average thickness of 4.65 m and the old roof is medium sandstone with an average thickness of 13.45 m. The comprehensive geological histogram is shown in Fig. 2. The thickness of the bedrock within the working face increases gradually from southwest to northeast, and the thickness of the thinnest part is approximately 60 m. The main water filling source in the mining area is the sandstone fissure aquifer of the 3 coal roof. This aquifer is composed of gray white quartz medium grained sandstone and fine grained sandstone, containing muddy inclusions or strips, with an average thickness of 45.8 m. The unit water inflow of the aquifer is 0.00001295 ~ 0.00821 L/s·m, and the permeability coefficient is 0.000599 ~ 0.016 m/d. The connectivity of the sandstone aquifer system in the roof of No.3 coal seam is poor, the water content is heterogeneous, and the scope of some water-rich areas is not clear.

In the cover rock of the working face, the bedrock is unstable and uneven in thickness, and the roof of the working face is covered with an aquifer. Therefore, if the fracture zone formed after mining of the working face leads to the aquifer, there is a risk of water inrush disaster. Therefore, a feasibility study should be carried out before mining the working face.

## Research methods

### Geophysical research on aquifer distribution

Using geophysical techniques such as the mine transient electromagnetic method and high-density three-dimensional electrical method^27–31^, the scope of the water-rich abnormal areas in the overlying aquifer of the 1318 working face was explored.

#### Transient electromagnetic method detection scheme

**Figure 3:**
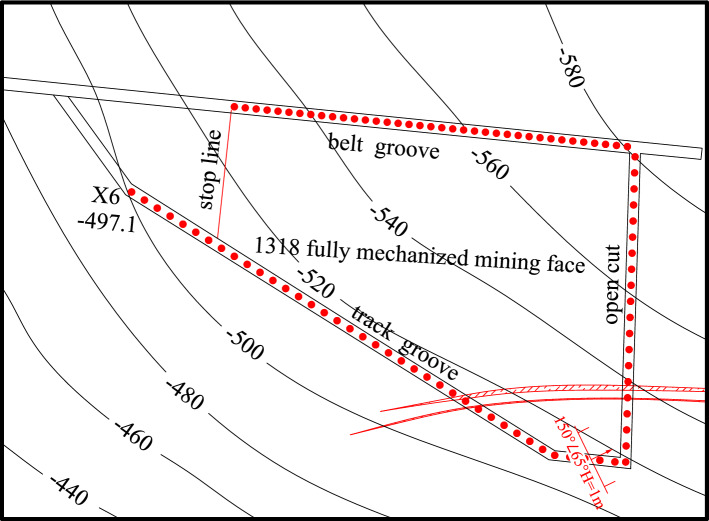
Schematic diagram of transient electromagnetic method detection.

**Figure 4:**
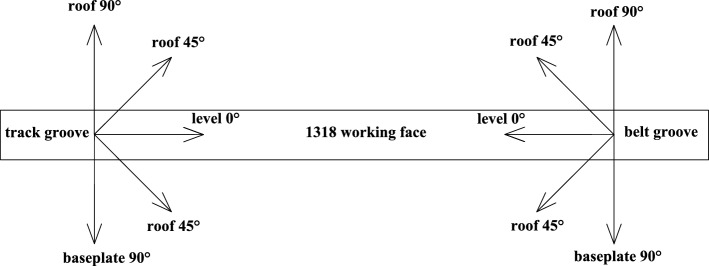
Detection direction diagram of the 1318 working face.

As shown in Figs. 3 and 4. The detection was carried out in the belt grooves, track grooves and cut-hole in the 1318 working face. The distance between adjacent detection positions was 10 m. A total of 37 detection positions were arranged from the stopping line to the cut-hole in the belt grooves, 38 detection positions were arranged from the X6 wire point to the cut-hole in the belt grooves, and 20 detection positions were arranged in the cut-hole. Among them, the roof direction of the working face was detected, and the roof detection direction was 45°, 90° upward and 45° downward. The two-dimensional resistivity profiles of each detection direction were carried out at two depths of 45 m and 60 m from the upper part of Coal Seam 3. The resistivity profiles of different angles of each survey line were comprehensively analyzed. The roof aquifer layer that threatens the mining of Coal Seam 3 was mainly analyzed, and a limestone resistivity bedding slice diagram was formed. The comprehensive analysis can obtain a more complete and accurate scope of the water-rich anomaly area of the overlying aquifer on the working face.

#### High-density electrical detection scheme

**Figure 5:**
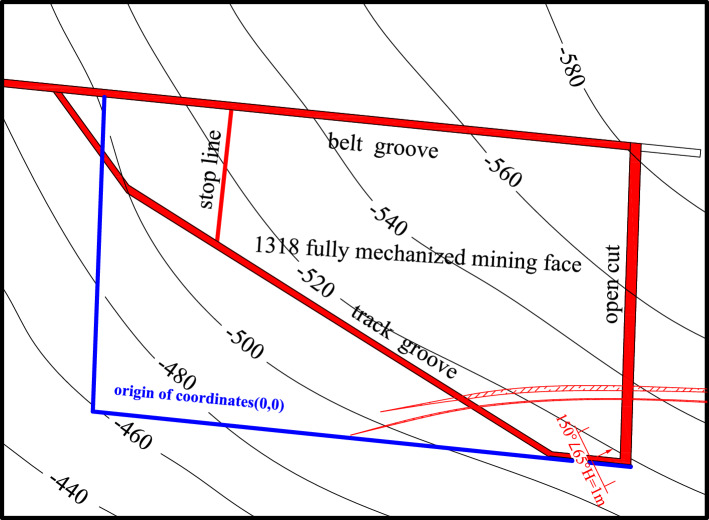
High-density electrical detection position layout diagram.

As shown in Fig. 5. Detection was carried out in the belt grooves, the track groove and the cut-hole of the 1318 working face, with 5 m spacing between adjacent measurement points. A total of 3 measuring lines were laid out, one measuring line was arranged in the belt grooves, with 72 measuring points, one measuring line was arranged in the track groove, with 88 measuring points, and one measuring line was arranged in the cut-hole, with 45 measuring points. Among them, the three-dimensional resistivity profile of each detection direction was divided into resistivity bedding slices of 30 m and 40 m from the upper part of Coal Seam 3. According to the reflection shape, range size and resistance value of the apparent resistivity isoline (chromatogram) in the apparent resistivity section map inverted by the high-density resistivity method, combined with geological and survey data, the scope of the water-rich anomaly area of the overlying aquifer on the working surface can be obtained more completely and accurately through the comprehensive analysis of the data.

### Drilling scheme of bedrock distribution characteristics

**Figure 6:**
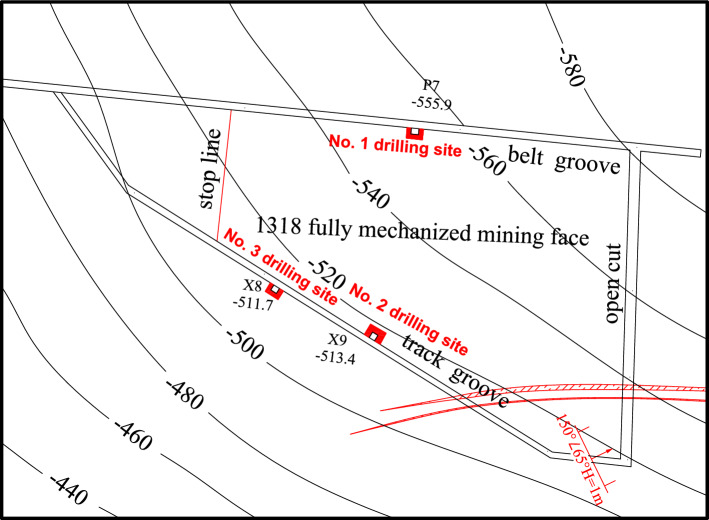
Drill hole distribution layout.

The bedrock drilling method was used to investigate the lithology, thickness and distribution of the roof aquifer and aquiclude of Coal Seam 3^32,33^. As shown in Fig. 6. The drilling chambers were designed with one rig chamber in the 1318 belt grooves and two rig chambers in the 1318 track groove. The chamber of drilling rig 1 is located 28 m behind point P7 of the 1318 belt groove, the chamber of drilling rig 2 is located 11.7 m before point X9 of the 1318 track groove, and the chamber of drilling rig 3 is located 10 m before point X8 of the 1318 track groove. Among them, the chamber of drilling rig 2 was constructed with 4 roof exploration and drainage boreholes, which were DT-1, DT-2, DT-3 and DT-4. The lithology of the boreholes was mainly sandstone. The drilling parameters are shown in Table 1. Through the comprehensive comparative analysis of drilling results, a more complete and accurate bedrock lithology, thickness and distribution of the overlying aquifer on the working face can be obtained.

**Table 1:** List of drilling parameters of the 1318 working face in Xiaoyun Coal Mine.

Hole number	Borehole location	Property	Drilling depth (m)	Vertical depth(m)	Bearing (°)	dip angle (°)
DT-1	No. 2 drilling rig chamber	Roof hole	119	27	13	13
DT-2		Roof hole	106	38	37	21
DT-3		Roof hole	125	47	107	22
DT-4		Roof hole	134	60	112	27

### Prediction of fracture zone development height

The development height of the fracture zone is the key factor for safe mining under the aquifer. The theoretical research on the development height of the fracture zone is the basis for evaluating the feasibility of safe mining in working faces^34–39^.

#### Empirical formula prediction

Table 2 is the formula for calculating the height of the water-conducting fracture zone widely used by Chinese coal mine field technicians currently, which is based on the empirical formula given in the Regulations on Building, Water Body, Railway and Main Roadway Coal Pillar Setting and Coal Pressure Mining^40^. The formula is obtained by statistical analysis of a large number of measured results. The overburden lithology is classified as "stiffness", "medium-hard", or "soft". For the "extremely soft" type, different formulas are used to calculate the height of the water-conducting fracture zone of the cover rock.

**Table 2:** Calculation formula for the height of the water-conducting fracture zone.

Lithologic characters	Computing formula(m)
stiffness	$H_{Ii} = \frac{100\sum M }{{1.2\sum M + 2.0}} \pm 8.9$
medium-hard	$H_{Ii} = \frac{100\sum M }{{1.6\sum M + 3.6}} \pm 5.6$
soft	$H_{Ii} = \frac{100\sum M }{{3.1\sum M + 5.0}} \pm 4.0$
Extremely soft	$H_{Ii} = \frac{100\sum M }{{5.0\sum M + 8.0}} \pm 3.0$

#### Theoretical prediction method based on rock stratum group

A large number of previous studies have shown that after the coal seam is mined, the movement of the overlying strata is a bending and sinking movement with the rock stratum group as a unit. Each stratum group is driven by a layer of hard rock at the bottom to coordinate the movement of the upper layers of weak rock. The movement combination of the rock stratum is determined by the strength factors (including lithology, thickness and elastic modulus) of each rock stratum. The upper rock stratum with low strength factors will move simultaneously with the lower rock stratum with high strength factors, and the subsidence curvature is the same. When a certain hard rock stratum has large subsidence, which leads to the formation of enough through cracks on the rock stratum surface to conduct water, the upper soft rock stratum controlled by it has the same subsidence as the rock stratum, and its anti-deformation ability is lower than the lower hard rock stratum, so its developed fractures also reach the penetration degree, so the rock stratum group will be classified into the category of water-conducting fractured zone at the same time. By analogy, when the subsidence of a hard rock stratum is not enough to form sufficient water-conducting fractures, the water-conducting fracture zone is highly developed until this layer of rock stratum. At this time, the rock stratum group above this layer belongs to the bending zone. Therefore, it can be seen that the water-conducting fracture zone of the overlying strata of the working face gradually develops upward in a ladder shape with the rock stratum group as the unit, and the fracture development of each stratum group is controlled by the movement of the lower hard rock formation. This understanding is different from the traditional concept of the formation of a water-conducting fracture zone. For two adjacent strata, whether they move together to form a stratum group or separately, the maximum curvature *ρ*_max_ of stratum settlement can be used to judge^41^.

When , the two strata are combined into a stratum movement.

When , the two strata move separately to form two stratum groups.

The maximum bending curvature of a rocking beam can be expressed as:

$$ \rho_{\max } = \alpha \frac{{\gamma L^{2} }}{{Em^{2} }} $$

where: *α* are coefficients determined by the supporting conditions of the rock beam; *L*—Limit span of rock beam; *E*—Elastic modulus of rock beam; *m*—thickness of stratum.

#### Analogy analysis of on-site measurement of adjacent working faces

The 1314 working face was adjacent to the 1318 working face, and the mining conditions of the 1314 working face are the same as those of the 1318 working face. The field measurement method of the adjacent working face was used to detect the 1314 working face on the spot, and the development height of the fracture zone of the 1318 working face was obtained through analysis. The field measurement principle and scheme design are as follows.

### Observation principle and method

#### Observation principle

**Figure 7:**
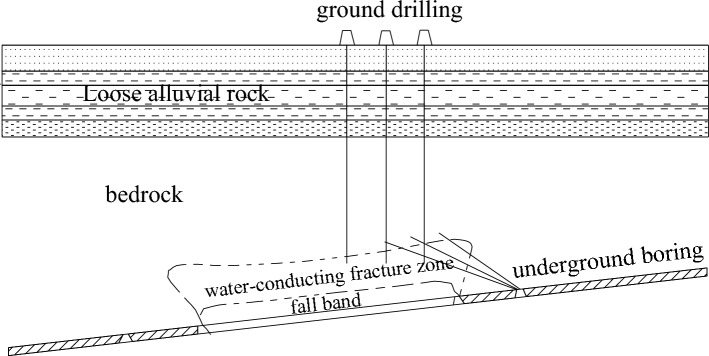
Height observation diagram of a water-conducting fracture zone in the underground borehole.

The development form of a water-conducting fracture zone in overlying strata after mining of the 1318 working face is detected and analyzed by using the observation method of water injection of the leakage of the underground inclined borehole. The principle of this observation method is to arrange a drilling field at a certain position around the underground mining face, drill an inclined borehole from the drilling field to the overburden water-conducting fracture zone above the goaf of the working face, and use the underground guide height observation instrument to observe the guide height, as shown in Fig. 7. The observation equipment is used to carry out segmented water injection observations on the borehole from bottom to top. According to the change rule of water injection leakage in different areas of the borehole, the development height, spatial boundary shape and other characteristics of the cover rock fracture zone can be analyzed and determined more clearly and accurately.

#### Observation method

**Figure 8:**
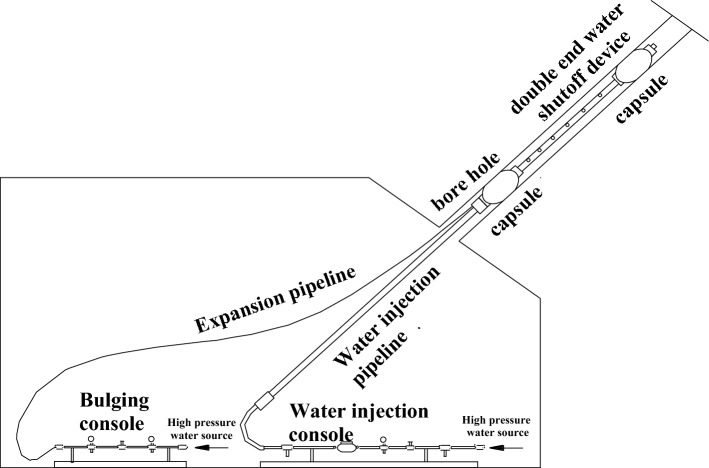
Test schematic diagram of the elevation observation instrument for the inclined borehole.

**Figure 9:**
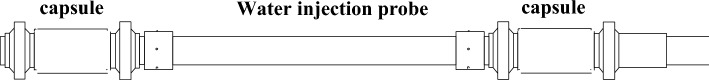
Structure diagram of the double-ended water shutoff device.

The downhole guide height observation instrument is arranged as shown in Fig. 8 and Fig. 9. The double-ended water shutoff device is composed of two expansion capsules and water injection probes. There are two connecting pipelines, the expansion pipeline and the water injection pipeline. The console is the expansion console and the water injection console. The expansion console, the expansion pipeline and the two capsules of the double-ended water shutoff are connected to form a capsule expansion and contraction pressure control system. The water injection console, the water injection pipeline and the water injection probe pipe of the double-end water shutoff device are connected to form a water injection observation system for observing the water conductivity of the rock stratum.

### The 1314 working face observation scheme design

#### Drilling site location design

**Figure 10:**
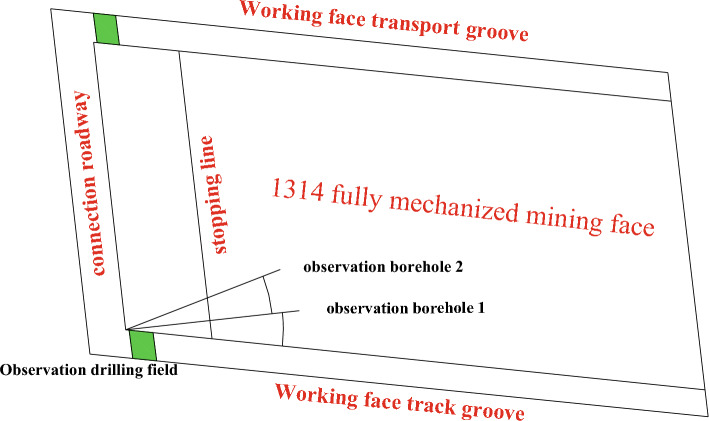
Observation borehole layout plan diagram.

According to the observation principle of the water-conducting fracture zone and the layout conditions of the roadway around the 1314 working face, the position of the observation drilling field in the water-conducting fractured zone is shown in Fig. 10. The observation position of the pilot height observation borehole was set near the intersection of the closed and contact roadways of the working face track groove. The straight-line distance between the drilling site and the stopping line of the working face was approximately 38.8 m. A total of two height observation boreholes, borehole 1 and borehole 2, were designed. The azimuth of observation borehole 1 was shifted to the left along the track groove by 12°, which was basically in the vertical direction with the stopping line of the working face. Borehole 2 continued to shift 15° to the left along borehole 1. It was used to observe the development of the overlying rock fracture zone in the direction of the working face.

#### Drilling parameter design

**Figure 11:**
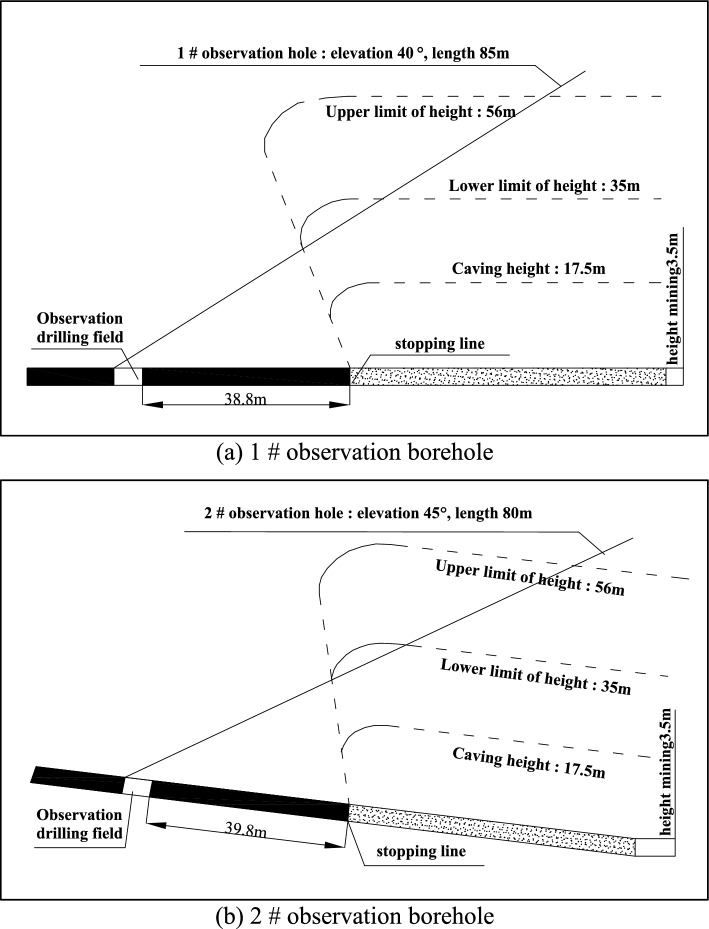
Height observation borehole layout profile.

The parameter design of the observation borehole is the key to field detection. The design parameters mainly include the dip angle and length of the borehole. According to the design requirements of the observation boreholes, the observation of the height of the water-conducting fracture zone in the overlying strata required two observation boreholes at the observation section. The boreholes were constructed from the vicinity of the intersection of the closed and contact lanes of the 1314 track groove to the stopping line of the 1314 working face. The elements of each borehole are shown in Table 3. The layout profile of the borehole for guided height observation is shown in Fig. 11.

**Table 3:** Working face conductor height observation drilling elements table.

Observation borehole	Dip angle	Length (m)	Aperture (mm)	Usage
1	40°	85	Φ94	Observation of height of the water-flowing fractured zone
2	45°	80	Φ94	Observation of height of the water-flowing fractured zone

## Research results

### Analysis of roof water-rich area

**Figure 12:**
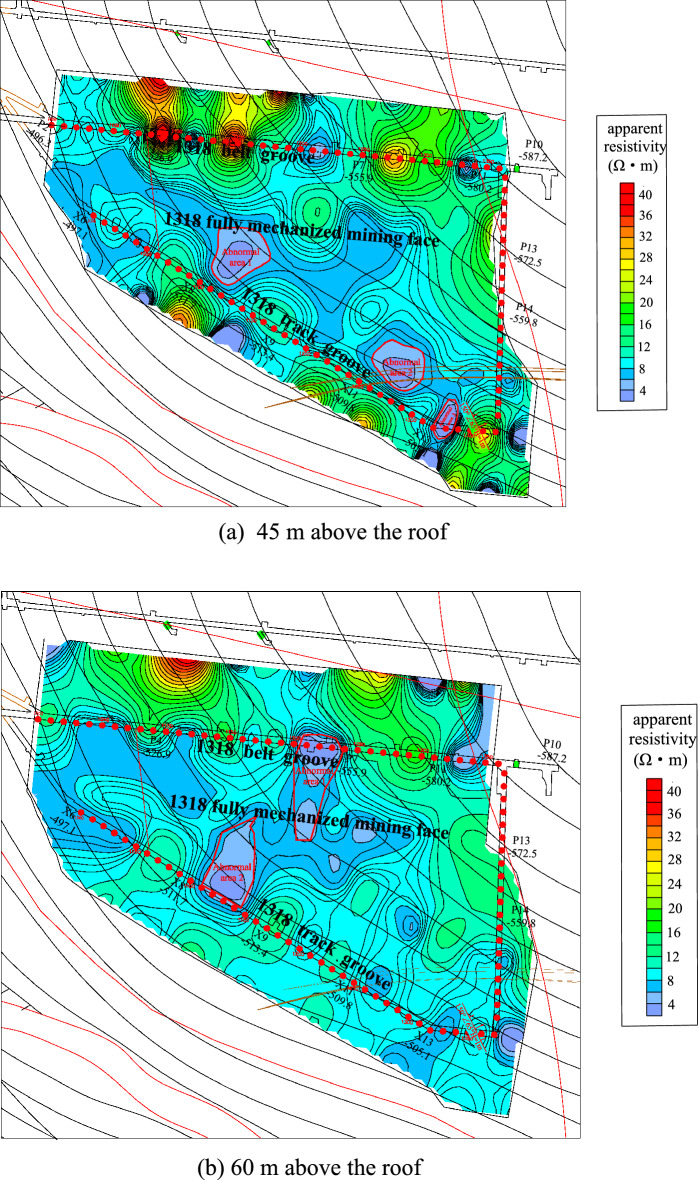
The 1318 Working face roof aquifer apparent resistivity bedding slice diagram and rich water distribution map.

According to the detection results of the transient electromagnetic method, Fig. 12 shows the anomaly map of apparent resistivity bedding slices at two depths of 45 m and 60 m from the roof of the working face. From the diagram, it can be seen that 45 m above the roof of Coal Seam 3: in the track groove pile number 1090 ~ 1140 m extended to the working face 60 m, the track groove pile number 1250 ~ 1290 m extended to the working face 50 m, and the track groove pile number 1320 ~ 1350 m extended to the working face 20 m. The apparent resistivity in the abnormal area was less than 4 Ω·m, and the water content of the aquifer was relatively strong. 60 m above the roof of the 3 coal seam: the track groove pile number 1100 ~ 11,150 m extended to the working face 70 m, and the belt groove pile number 1200 ~ 11,240 m extended to the working face 70 m. The apparent resistivity in the abnormal area was less than 4 Ω·m, and the water content of the aquifer was relatively strong.

**Figure 13:**
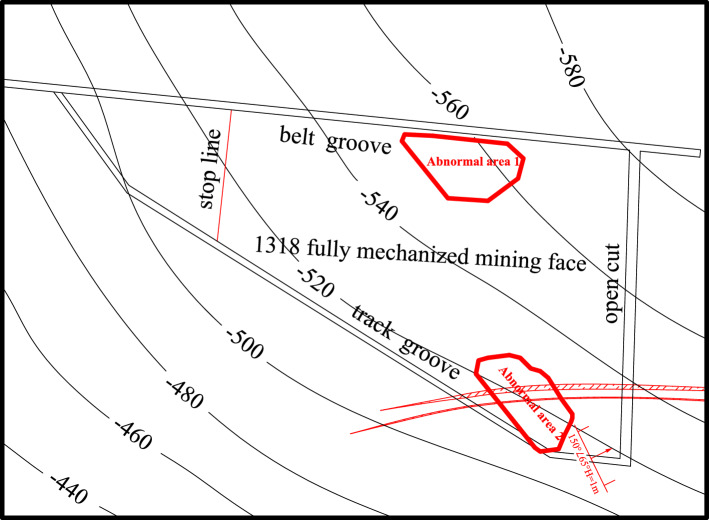
Roof abnormal area of the working face.

In combination with the results of transient electromagnetic exploration, bedrock exploration results and roof watering during roadway excavation, some abnormal areas were verified. Two abnormal areas were drawn above the roof in the working face, as shown in Fig. 13. Among them, the vertical range of abnormal area 1 is 45–60 m from the roof, with an area of 3334 m^2^; the vertical range of abnormal area 2 is 30–60 m from the roof, with an area of 2520 m^2^.

**Figure 14:**
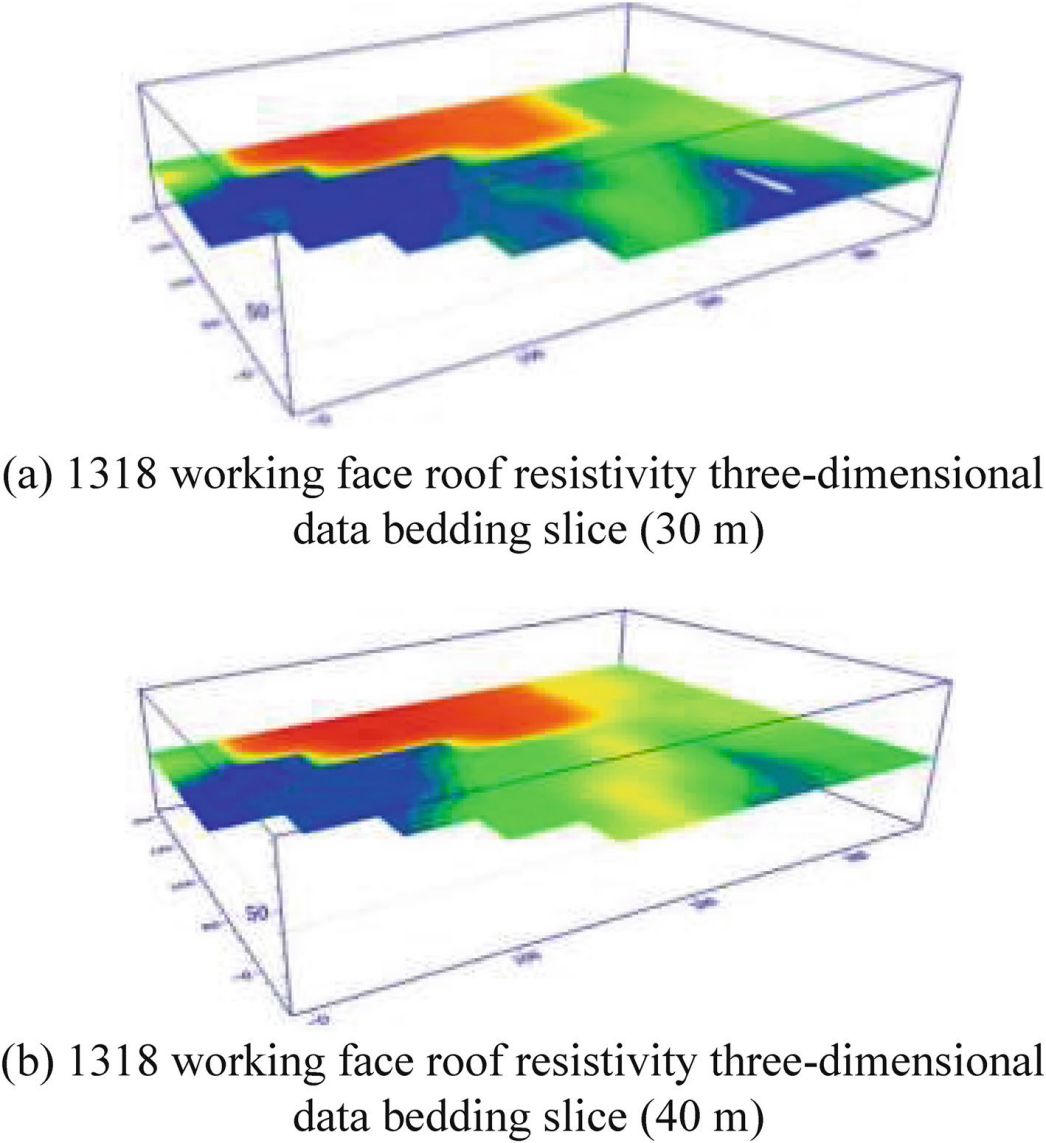
Three-dimensional bedding slice data of the 1318 working face roof resistivity.

According to the analysis of the high-density electrical method results, Fig. 14 shows the high-density electrical method of the geophysical exploration method to detect the working face roof 30 m and 40 m resistivity three-dimensional bedding slice anomaly map. Comparing the 30 m bedding slice above the roof with the 40 m bedding slice above the roof, it can be seen that the resistivity of the bedding slice 30 m above the roof was the lowest, the resistivity of the abnormal area was less than 2 Ω·m, and the water content of the aquifer was relatively strong.

**Figure 15:**
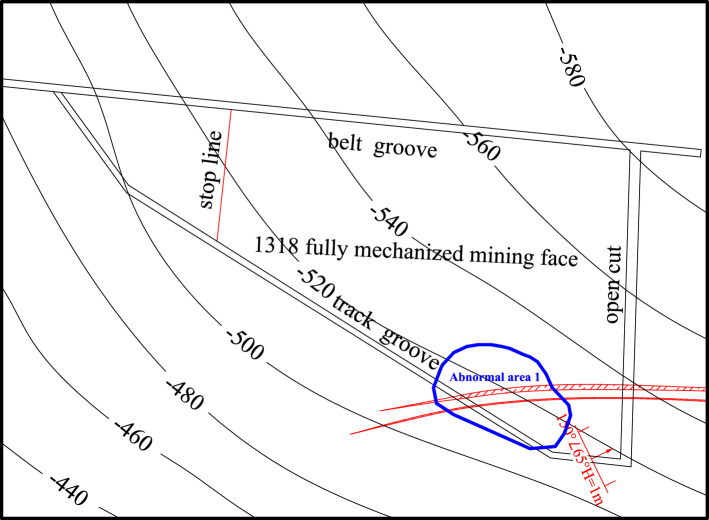
Water-rich distribution of the roof sandstone aquifer in the 1318 working face.

Combined with the structural distribution characteristics and hydrogeological conditions of the working face and the results of advanced exploration and drainage in the process of roadway excavation, the water-rich condition of the sandstone aquifer on the roof of the working face is inferred and explained (see Fig. 15). A total of 1 abnormal area was explained in the whole area, with a total area of approximately 3306 m^2^.

Based on the above geophysical exploration results, two abnormal areas were found in the whole area. The vertical range of abnormal area 1 was 45–60 m from the roof, with an area of 3334 m^2^. The vertical range of abnormal area 2 was 30–60 m from the roof, with an area of approximately 2913 m^2^. The water-bearing condition of the roof aquifer of the coal seam in Working Face 1318 was clarified during this survey, and the water-bearing property of the roof aquifer was relatively weak.

### Bedrock thickness and structure analysis

**Figure 16:**
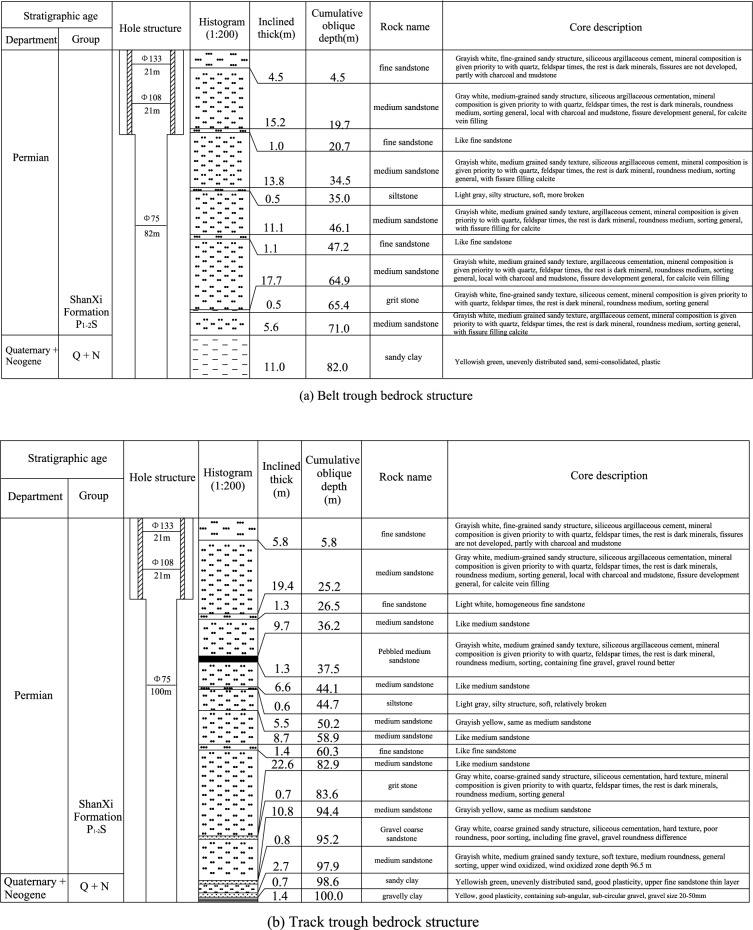
Typical borehole histogram.

Combined with the drilling results, some (TC1-1, TC2-1) drill hole columnar sections were drawn through comprehensive analysis. According to the analysis in Fig. 16, it was concluded that the types of bedrock in the range of the 1318 working face are mainly medium sandstone, fine sandstone, siltstone, coarse sandstone, gravelly medium sandstone and gravelly coarse sandstone, mainly medium sandstone. The cumulative thickness of medium sandstone accounted for approximately 89% of the detected bedrock thickness. Except for the fact that the thickness of fine sandstone at the direct roof of the coal seam was 4.65 m, the average thickness of the other types of rock strata was relatively small, approximately 1 m, which can be regarded as the interlayer of medium sandstone.

**Figure 17:**
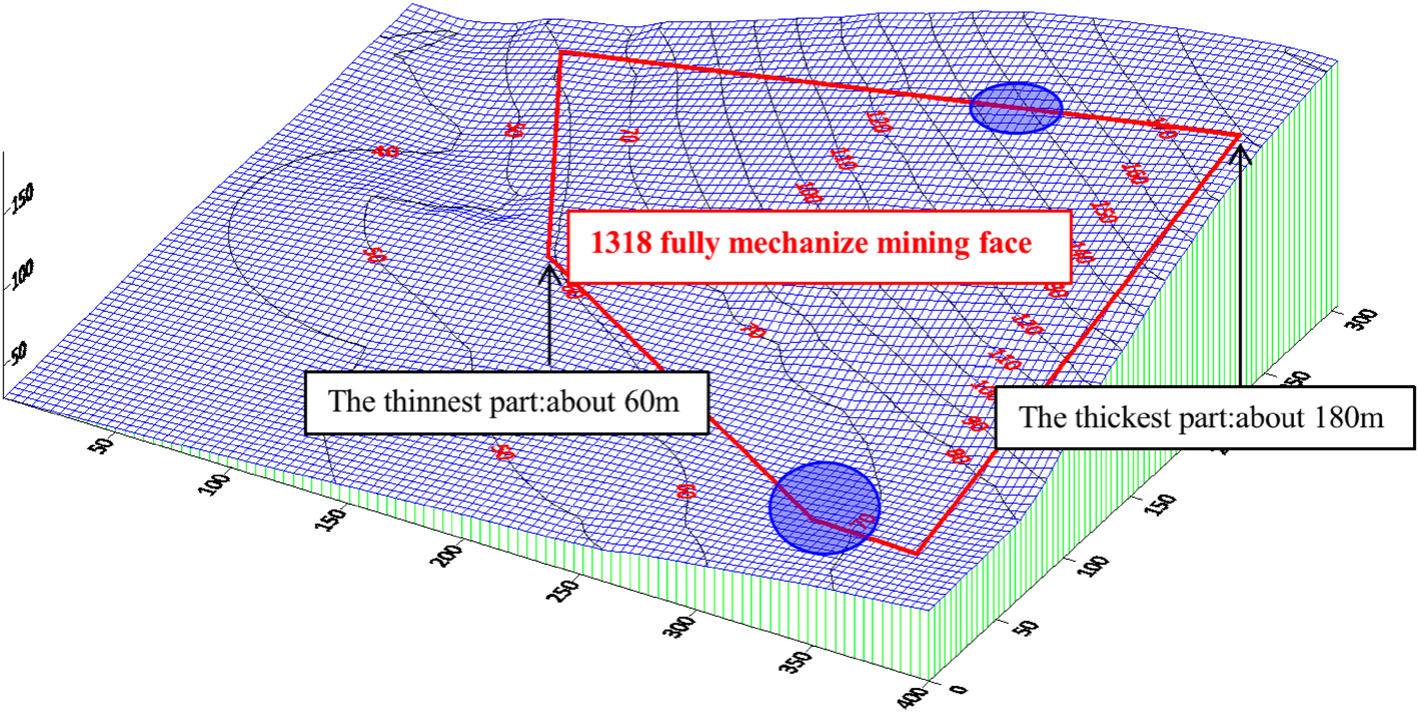
Stereogram of bedrock thickness.

According to the drilling data, the bedrock thickness stereogram was drawn after analysis, as shown in Fig. 17. The bedrock thickness in the range of the 1318 working face increases gradually from southwest to northeast. The thinnest part was located at the intersection of the stopping line and track groove, and the thickness was approximately 60 m. The thickest part was located at the intersection of the cut-hole and belt groove, and the thickness was approximately 180 m. The bedrock thickness in the mining range of the whole 1318 working face was greater than the 55 m required by the safety mining specification.

### Analysis of the development height of the fracture zone

#### Calculation results of the empirical formula

According to the analysis of the surrounding rock structure and mechanical characteristics of the 1318 working face in Xiaoyun Coal Mine, the overlying strata of Coal Seam 3 were mainly siltstone and medium fine sandstone, and the uniaxial compressive strength of rock was above 50 MPa. The overall comprehensive analysis showed that the overlying strata of the working face belonged to the category of medium hard rock stratum. Using the empirical formula corresponding to the “Empirical formula prediction” section, taking the maximum mining thickness of the Coal Seam 3 1318 working face as M = 3.3 m, the calculation results of the development height of the falling zone and the water-conducting fracture zone after the mining of the working face are shown in Table 4.

**Table 4:** Prediction results of the falling zone and water-conducting fracture zone.

Lithologic characters	Caving zone height/m	Height of fractured water-conducting zone/m
Medium-hard	7.4 ~ 11.8	31.4 ~ 42.6

#### Theoretical prediction results

**Figure 18:**
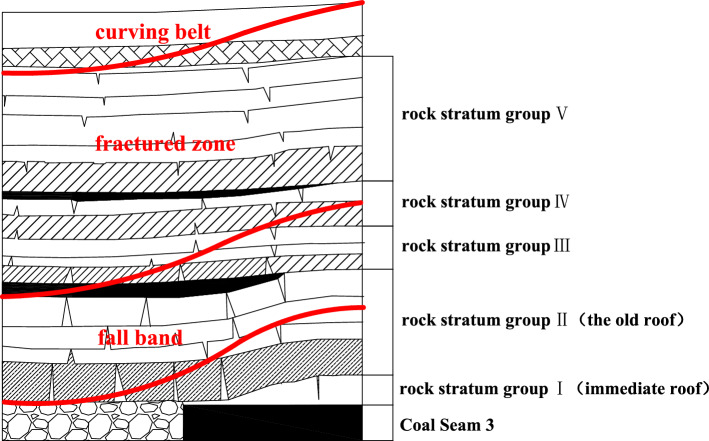
Cover rock combination and fracture characteristics.

According to the analysis of the characteristics of rock stratum movement, the composite structure of the overlying strata of Coal Seam 3 at the track groove side of Working Face 1318 in Xiaoyun Coal Mine was determined. Based on the theoretical prediction formula corresponding to Section “Theoretical prediction method based on rock stratum group”, the calculation and analysis were carried out one by one. The overlying bedrock range of the 1318 working face can be divided into five strata groups with alternating soft and hard combinations. As shown in Table 5. The overall thickness of rock stratum Group I was 4.1 m, which directly covered Coal Seam 3 of the working face and belongs to the direct roof category of Coal Seam 3. After the coal seam of the working face was mined, the rock stratum group gradually collapsed with the gradual increase in the mining space and accumulated in the goaf. The overall thickness of strata group II was 22.42 m. Because of its large overall thickness and high strength, the bending fracture would drive the overlying three coal-rock masses in the strata group to move at the same time, forming a composite old roof rock structure. The characteristic law of movement and failure of the rock stratum group is shown in Fig. 18.

**Table 5:** The 1318 working face cover rock combination structure division.

Rock stratum number	Rock stratum name	Lamination thickness/m	Vertical height from coal seam/m	Rock grouping structure	Rock group thickness/m
17	Gravelly clay	0.99	70.71	Loose layer	1.48
16	Sandy clay	0.49	69.72		
15	Medium sandstone	1.91	69.23	Rock stratum group V	26.59
14	Gravel coarse sandstone	0.57	67.32		
13	Medium sandstone	7.64	66.75		
12	Grit stone	0.49	59.11		
11	Medium sandstone	15.98	58.62		
10	Fine sandstone	0.99	42.64	Rock stratum group IV	7.14
9	Medium sandstone	6.15	41.65		
8	Medium sandstone	3.89	35.50	Rock stratum group III	8.98
7	Siltstone	0.42	31.61		
6	Medium sandstone	4.67	31.19		
5	Pebbled medium sandstone	0.92	26.52	Rock stratum group II (upper roof)	22.42
4	Medium sandstone	6.86	25.60		
3	Fine sandstone	0.92	18.74		
2	Medium sandstone	13.72	17.82		
1	Fine sandstone	4.10	4.10	Rock stratum group I (immediate roof)	4.1
	Coal Seam 3	3.30	0

According to the analysis of structural characteristics of the cover rock in Xiaoyun Coal Mine, because the cover rock of Coal Seam 3 1318 working face was mostly composite rock structure, the dynamic movement of rock stratum would show obvious incoordination. According to the classical theory of rock pressure and the calculation and analysis of rock beam mechanical movement, the overlying strata were classified by strata combination. After the coal seam of the working face was mined, the movement form of the overlying strata should be the bending subsidence movement with the rock stratum group as the unit. Each stratum group was controlled by the supporting layer with a large thickness and high strength at the lower part, which drove the synchronous and coordinated movement of the upper weak rock layers, and the subsidence curvature was the same. When the lowermost supporting layer was bent and broken, the overlying soft rock strata would move and break at the same time.

#### Field measurement results

Reliable observation data have been obtained from the two boreholes of Working Face 1314. According to the data of these two boreholes, the development height and shape of the overlying water-conducting fracture zone of the working face can be accurately determined.

##### Analysis of borehole observation results

**Figure 19:**
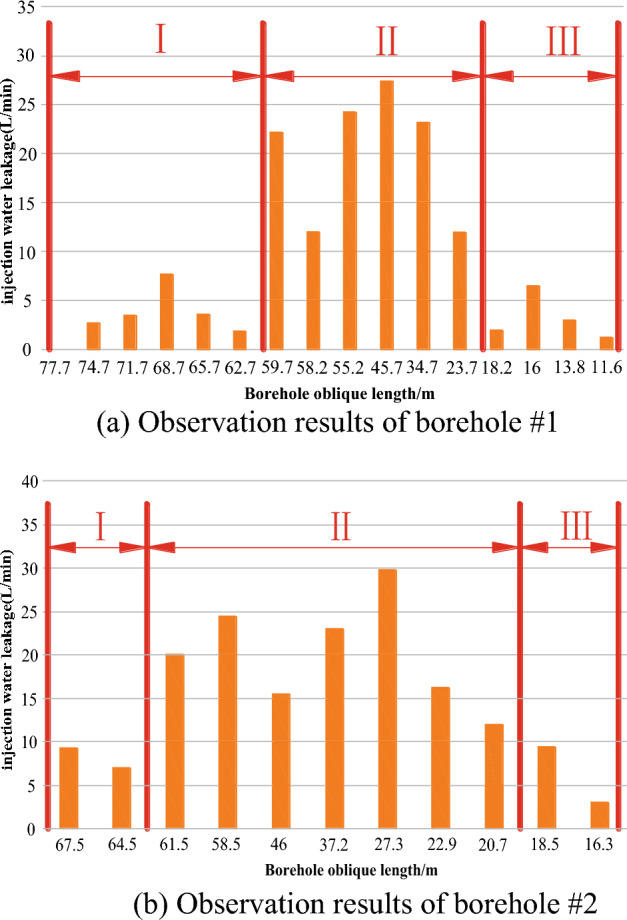
Borehole observation results.

According to the field observation data, the permeability diagram of each section of the rock stratum in hole #1 is drawn as shown in Fig. 19. The water injection leakage in each section of the borehole had obvious segmentation characteristics, and there was a great difference in the water injection leakage in each section, indicating that the whole borehole passes through different rock fracture development sections, which also verified the rationality of the borehole design. According to the observation sequence from bottom to top in the borehole, the water injection leakage of each section of the rock stratum in the borehole was summarized and analyzed as follows: in the borehole area of Section I, the inclined length of the borehole was 77.7 ~ 59.7 m, the vertical height was 49.9 ~ 38.4 m, the water seepage of the rock stratum was 0 ~ 7.6 L/min, the permeability of the whole section of the rock stratum was less than 10 L/min, and the permeability of the rock stratum was small, indicating that the second opening in the rock stratum was not developed, and the rock stratum was mainly the primary opening. Because the permeability of the rock stratum was small, the borehole in this area was outside the development range of the water-conducting fracture zone, and the borehole had not yet entered the fracture zone area. In the borehole area of section II, the inclined length of the borehole was 59.7 m ~ 23.7 m, the vertical height was 38.4 m ~ 15.2 m, and the water seepage volume of the rock stratum increased sharply to 12.0 ~ 27.3 The water seepage volume of the rock stratum was large, indicating that the borehole had penetrated into the fracture zone at this time. In this area, the rock stratum was greatly affected by mining. The secondary opening produced by the sinking and bending of the rock stratum was relatively developed, and the connectivity between the openings was good. Therefore, the water injection leakage of the borehole was large, and the overall water conductivity of the rock stratum was strong. In the borehole area of section III, the inclined length of the borehole was 23.7 m ~ 11.6 m, the vertical height was 15.2 m ~ 7.5 m, the water seepage of the rock stratum was 1.2 ~ 6.5 L/min, the permeability of the whole section of the rock stratum was less than 10 L/min, and the permeability of the rock stratum was small, indicating that the second opening in the rock stratum was not developed, and the rock stratum was mainly the primary opening. Because the permeability of the rock stratum was small, the development height of the fracture zone was not affected here, and it was basically in the category of a bending subsidence zone.

According to the inflection point of the change in the water injection leakage of the rock stratum in the three-section drilling area, the development height of the water-conducting fracture zone obtained from observation hole #1 was approximately 38.4 m. In the same way, it can be concluded that the development height of the water-conducting fracture zone obtained from observation hole #2 was approximately 39.5 m.

Based on the above analysis, the observation results of the drilling observation section were summarized: hole #1: H (1) = 38.4 m, hole #2: H (2) = 39.5 m. The observation results of the two boreholes were relatively close. To ensure a high safety factor, the maximum value was taken as the final result. Therefore, according to the field-measured data, it was finally determined that the field-measured result of the development height of the water-conducting fracture zone in the 1314 working face of the Xiaoyun Coal Mine was 39.5 m.

Based on the investigation and analysis of the mining thickness of the coal seam in the working face near the observation location, the mining thickness was taken as 3.5 m, so the crack mining ratio parameter T of the 1314 working face of Xiaoyun Coal Mine can be obtained: T = 39.5/3.5 = 11.29.

**Figure 20:**
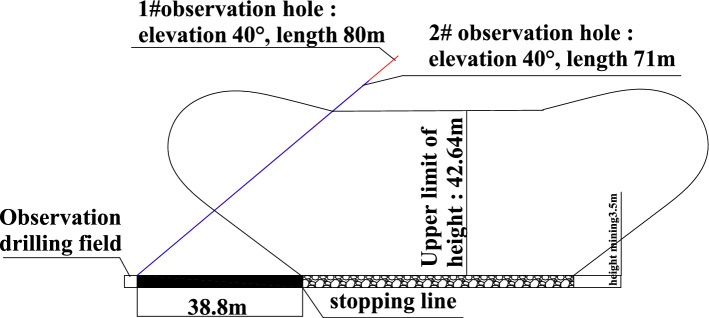
Fitting curve of development morphology of overburden fracture zone in 1314 working face.

At the same time, according to the observation data points, the shape of the overlying water-conducting fracture zone on the 1314 working face of Xiaoyun Coal Mine is shown as a relatively regular "saddle-shaped" distribution. Due to the differences in design dip angle and azimuth angle, the boreholes 1 # and 2 # pass through the gentle and peak areas in the upper part of the "saddle shaped" fracture zone, respectively. The observation results of 2 # boreholes are the largest, proving that they precisely pass through the maximum height area of the fracture zone development. The fracture zone morphology plotted by the drilling site has a very high similarity, as shown in Fig. 20.

According to the crack-mining ratio of the 1314 working face after mining, the measured height of the water-conducting fracture zone measured in the 1318 working face was 31.61 m, and the corresponding height guiding mining thickness ratio was 11.29 m.Based on the development morphology of the water conducting fracture zone in the 1314 working face, it is analogically analyzed that under the same geological conditions, the development morphology of the water conducting fracture zone in the 1318 working face presents a "saddle shaped" shape.

##### Summary analysis of observation results

According to the empirical formula of the Regulations on Building, Water Body, Railway and Main Roadway Coal Pillar Setting and Coal Pressure Mining, the theoretical prediction method based on rock stratum group calculation and field measurement, the predicted values of the water-conducting fracture zone were obtained, as shown in Table 6. Through the comparative analysis of the three methods, for the sake of safety, the maximum height of the water-conducting fracture zone was 42.64 m, and the corresponding height-mining thickness ratio was 12.92.

**Table 6:** Estimated height of the water-conducting fracture zone in working face 1318.

	Expected by 'norm'	Theoretical prediction of rock group calculation	Field measurement prediction
Water-conducting fracture zone height	31.4 ~ 42.6	35.5 ~ 42.64	31.61
Conductor height mining thickness ratio	9.5 ~ 12.9	10.76 ~ 12.92	11.29

## Working face mining safety discussion and analysis

**Figure 21:**
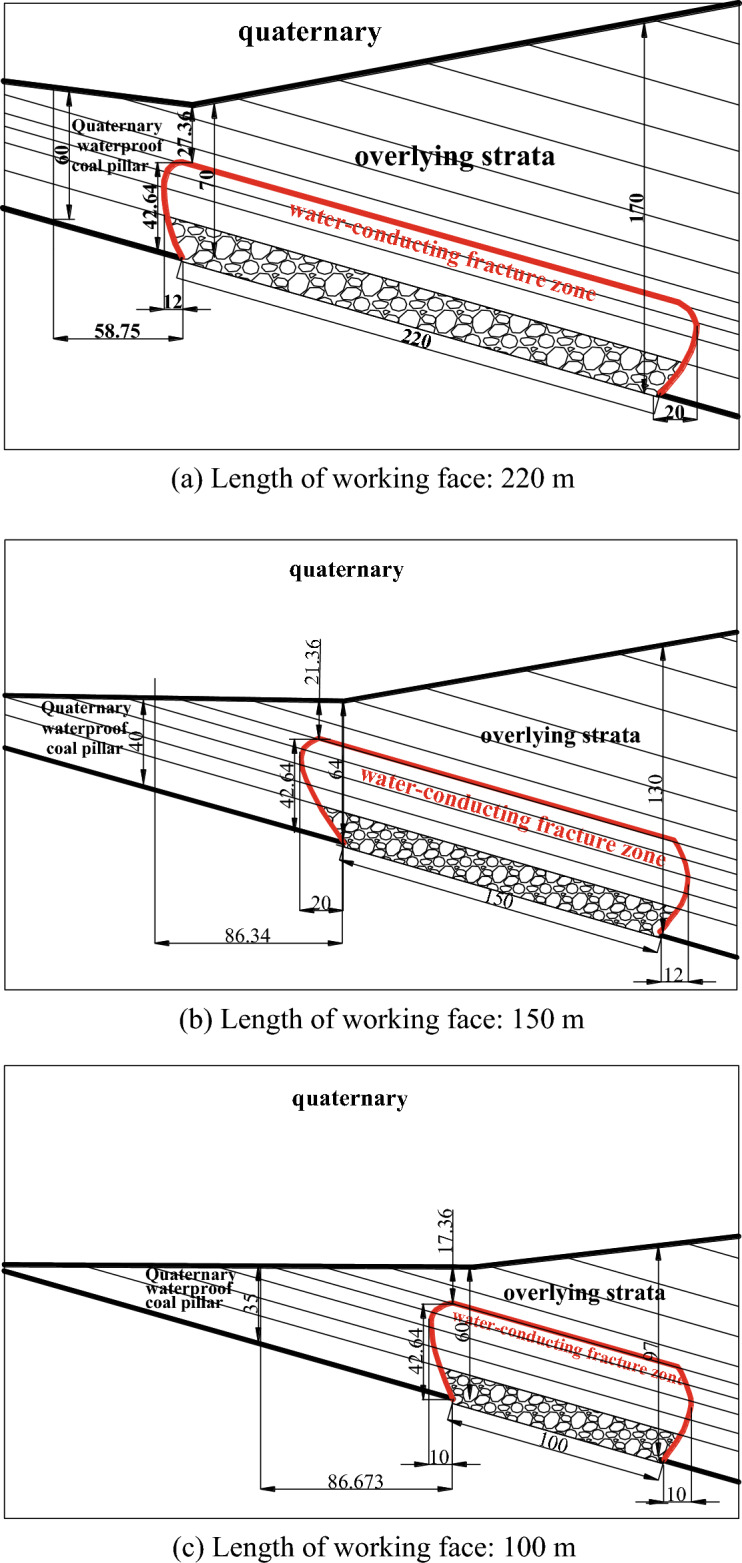
Prediction of the water-conducting fracture zone of the working face.

According to the result analysis of the geophysical prospecting method, the vertical range of anomaly zone 1 was 45–60 m from the roof, with an area of 3334 m^2^; the vertical range of abnormal area 2 was 30–60 m from the roof, with an area of approximately 2913 m^2^. According to the analysis of drilling data, it was concluded that the thinnest bedrock in the range of the 1318 working face was located at the intersection of the stopping line and the track groove, with a thickness of approximately 60 m. The above abnormal areas were all dangerous areas of this mining. With the mining of the 1318 working face, the length of the working face was gradually shortened. According to the distribution law of bedrock thickness, as shown in Fig. 21, the prediction of the water-conducting fracture zone of the working face with different working face lengths was made. With the advance of the working face, the range of the water-conducting fracture zone was continuously reduced.

**Figure 22:**
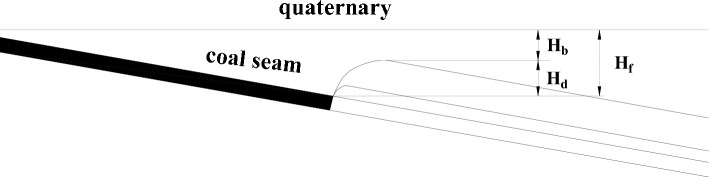
Mechanical model of water prevention coal (rock) pillar stratum structure.

The stratum structure mechanics model of the safety water prevention coal(rock) pillar is shown in Fig. 22, in which the height of the water-conducting fractured zone is Hd, the thickness of the protective layer is Hb, and the vertical height of the safety water prevention coal(rock) pillar is Hf. Through bedrock exploration, it was determined that the Quaternary bottom clay layer was greater than the maximum mining height of the 1318 working face, and the bedrock was basically medium sandstone, which belongs to medium hard rock. Therefore, as shown in Table 7, the thickness of the protective layer Hb was taken for mining under the Quaternary system of this working face was 3A, which was 3 × 3.3 = 9.9 m. Through comprehensive calculation, it was concluded that the safety water prevention coal(rock) pillar Hf under the Quaternary alluvium of the working face was the sum of the height of the water-conducting fracture zone Hd and the thickness of the protective layer Hb, which was 42.64 + 9.9 = 52.6 m. The safety water prevention coal(rock) pillar designed in the mine safety section was 55 m, and the safety water prevention coal(rock) pillar left in the actual mining range was greater than 60 m. Therefore, the extraction of the 1318 working face will not be affected by the Quaternary impact layer water.

**Table 7:** Thickness of waterproof safety coal rock pillar protective layer.

Cover rock lithology	Cohesive soil thickness at the bottom of loose layer is greater than cumulative mining thickness	Cohesive soil thickness at the bottom of loose layer is less than cumulative mining thickness	Loose layer full thickness	Noncohesive soil layer at the bottom of loose layer
Stiffness	4A	5A	6A	7A
Medium-hard	3A	4A	5A	6A
Soft	2A	3A	4A	5A
Extremely soft	2A	2A	3A	4A

Note: 1. $A = \frac{\sum M }{n}:\;\sum M$—Cumulative mining thickness; n—layer number; 2. Suitable for gently inclined (0° ~ 35°), inclined (36° ~ 54°) coal seams.

## Conclusion

1. Under the complex bedrock structure, the safe mining of the irregular working face was affected by many factors, such as the scope of the water-rich area, the thickness and structure of the cover rock, and the development height of the water-conducting fracture zone. Studying the influence of the above factors on safe mining under the aquifer of the working face played an important guiding role in the feasibility analysis of safe mining under the working face of a complex bedrock aquifer.
2. The scope of the water-rich area was determined by the geophysical exploration method, and the vertical range of abnormal area 1 was 45–60 m from the roof, with an area of 3334 m^2^. The vertical range of abnormal area 2 was approximately 2913 m from the roof, with an area of approximately 2913 m^2^. It provides a scientific basis and data support for accurately judging safe mining under an aquifer with a complex bedrock working face.
3. The thickness and structure of the cover rock were determined by the drilling method. Through analysis of drilling data, it was concluded that the thinnest part of the 1318 working face was located at the intersection of the stopping line and belt groove, with a thickness of approximately 60 m, and the thickest part was located at the intersection of the cut-hole and belt groove, with a thickness of approximately 180 m. The bedrock in the range of the 1318 working face was low in the southwest and high in the northeast.
4. According to the three methods of the empirical formula, rock stratum group theory prediction and field detection analogy analysis of the adjacent working face, the maximum height of fracture zone development after working face mining was 42.64 m.
5. Based on the comparative analysis of the results of the scope of the water-rich area, the thickness and structure of the cover rock, and the development height of the water-conducting fractured zone, the mining dangerous abnormal area was determined, and the size of the water prevention coal(rock) pillar was 52.6 m, which was smaller than the safety water prevention coal(rock) pillar actually left in the mining area.
6. From the perspective of water safety prevention, the working face has the conditions for safe mining on the premise of good mining monitoring and emergency measures during the mining process. The research conclusion provides important safety guidance significance for the mining of similar mines.

## Data Availability

All data generated or analysed during this study are included in this published aricle.
